# Mycophenolate Mofetil-Induced Aphthous Stomatitis After Kidney Transplant: A Clinical Case Report

**DOI:** 10.7759/cureus.50811

**Published:** 2023-12-19

**Authors:** Asma Almeslet

**Affiliations:** 1 Department of Oral Maxillofacial Surgery and Diagnostic Sciences, Riyadh Elm University, Riyadh, SAU

**Keywords:** oral mucosal lesions, case report, kidney transplant, aphthous stomatitis, mycophenolate mofetil

## Abstract

Aphthous stomatitis, with frequent and painful aphthous ulcers (also called canker sores or simply oral ulcers) on the non-keratinized oral mucous membranes, is often a cutaneous complication in kidney transplant recipients. Mycophenolate mofetil is a drug that is part of an immunosuppressive regimen in kidney transplant recipients, along with corticosteroids and calcineurin inhibitors. Mycophenolate mofetil, the triggering agent for mouth ulcers in kidney transplant recipients, has been communicated in scientific literature. Herein, we report a case of multiple painful oral ulcerations secondary to mycophenolate mofetil in a 23-year-old female kidney transplant recipient. The oral ulcers resolved immediately after discontinuation of the mycophenolate mofetil. Withdrawal of mycophenolate mofetil or switching to other medicine must be considered when a patient with a kidney transplant history complains of oral ulcers and a negative workup for other etiologies.

## Introduction

Organ transplant recipients are at high risk of infection owing to immunosuppression, which can be expressed as the development of oral ulcers. It has been reported that over 60% of kidney transplant recipients develop oral ulcers [1]. Oral diseases are occasionally ignored; however, they can significantly affect the quality of life and often transform into chronic systemic issues like nutritional deficiency and weight loss. Therefore, it is essential for physicians to appropriately recognize the cause of oral ulcers and offer immediate treatment [2]. Nevertheless, precise and immediate diagnosis is often challenging due to the etiological factors of oral ulcers in kidney transplant recipients, i.e., these comprise pathogens like bacteria, viruses, and fungi [3]. In fact, the list of differential diagnoses also contains drugs-induced oral ulcers, such as those associated with mycophenolate mofetil [2].

Mycophenolate can weaken the body's immune system and may decrease the ability to fight infection. In kidney transplant recipients, it is considered a drug of an immunosuppressive regimen along with corticosteroids and calcineurin inhibitors. While mycophenolate mofetil is well-tolerated among kidney transplant recipients, it has few adverse effects mostly related to the gastrointestinal system, like nausea, vomiting, diarrhea, dyspepsia, stomach ache, and gastroesophageal reflux [4]. Bone marrow suppression is yet another adverse effect [5]. Apart from that, a handful of cases of mycophenolate mofetil-induced oral ulcers have been reported in the scientific literature [2,6-8].

Herein, we report a case of multiple painful oral ulcerations secondary to mycophenolate mofetil in a 23-year-old female kidney transplant recipient. The oral ulcers resolved immediately after discontinuation of the mycophenolate mofetil.

## Case presentation

A 23-year-old single female, a student at university, was referred to the dental clinic for evaluation of multiple painful oral ulcerations. The patient’s medical history revealed end-stage renal disease (ESRD). The patient had a renal problem for the past five years. In February 2022, she underwent a living donor kidney transplantation from her brother. She received the following immunosuppressive medicines: mycophenolate mofetil (2 g/day), tacrolimus (1 mg/day), and methylprednisolone (5 mg/day) (Table 1). The patient did not have any other systemic disease or allergy.

**Table 1:** Medications at the time of admission. PO: per oral

Medication name	Dosing	Duration
Mycophenolate mofetil	2 g	Every 12 hours PO
Tacrolimus	1 mg	Every 12 hours PO
Methylprednisolone	5 mg	Daily PO
Fluconazole	200 mg	Daily PO
Nystatin	100,000 units/mL	Every 6 hours oral rinse/expectorant

**Figure 1:**
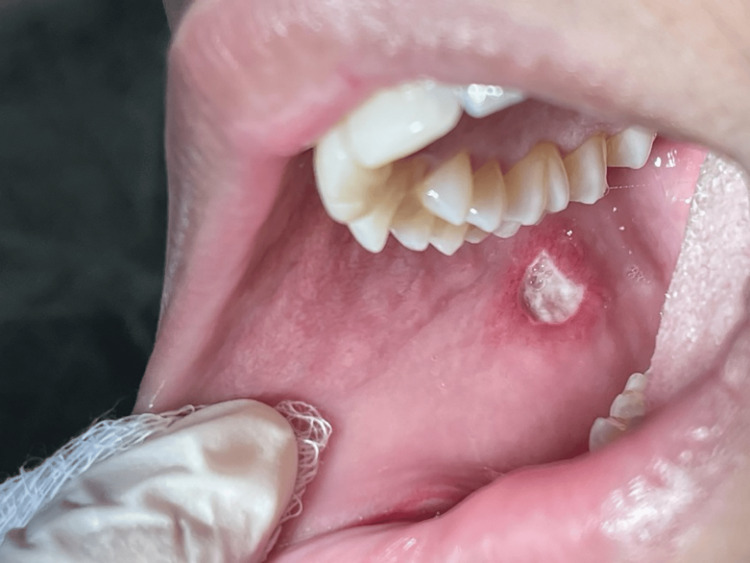
Aphthous ulcer with surrounding edema and erythema on buccal mucosa.

**Figure 2:**
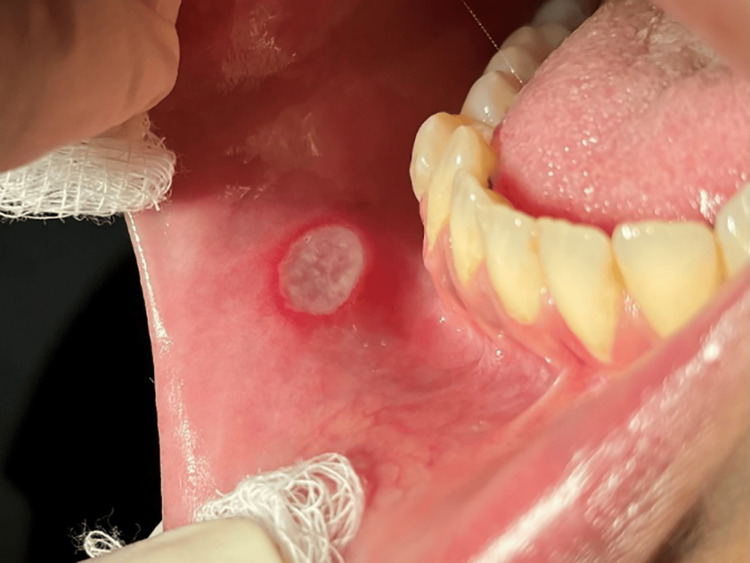
Aphthous ulcer with surrounding edema and erythema on labial mucosa.

After three months (May 2022) of kidney transplantation, she noticed three painful oral ulcers on the side of the tongue, buccal, and labial mucosa (approximately 1x1 cm). The patient reported for treatment after the second episode in June 2022. No history of any similar oral ulcers before transplantation was reported. She further complained of difficulty in swallowing, decreased intake, and weight loss without fever. She was treated with fluconazole for three weeks with a slight improvement in the oral ulcer. On examination in June 2022, a provisional diagnosis of two major aphthous ulcers was given, with surrounding edema and erythema present on his buccal mucosa (Figure 1) and another on the labial mucosa (Figure 2).

**Figure 3:**
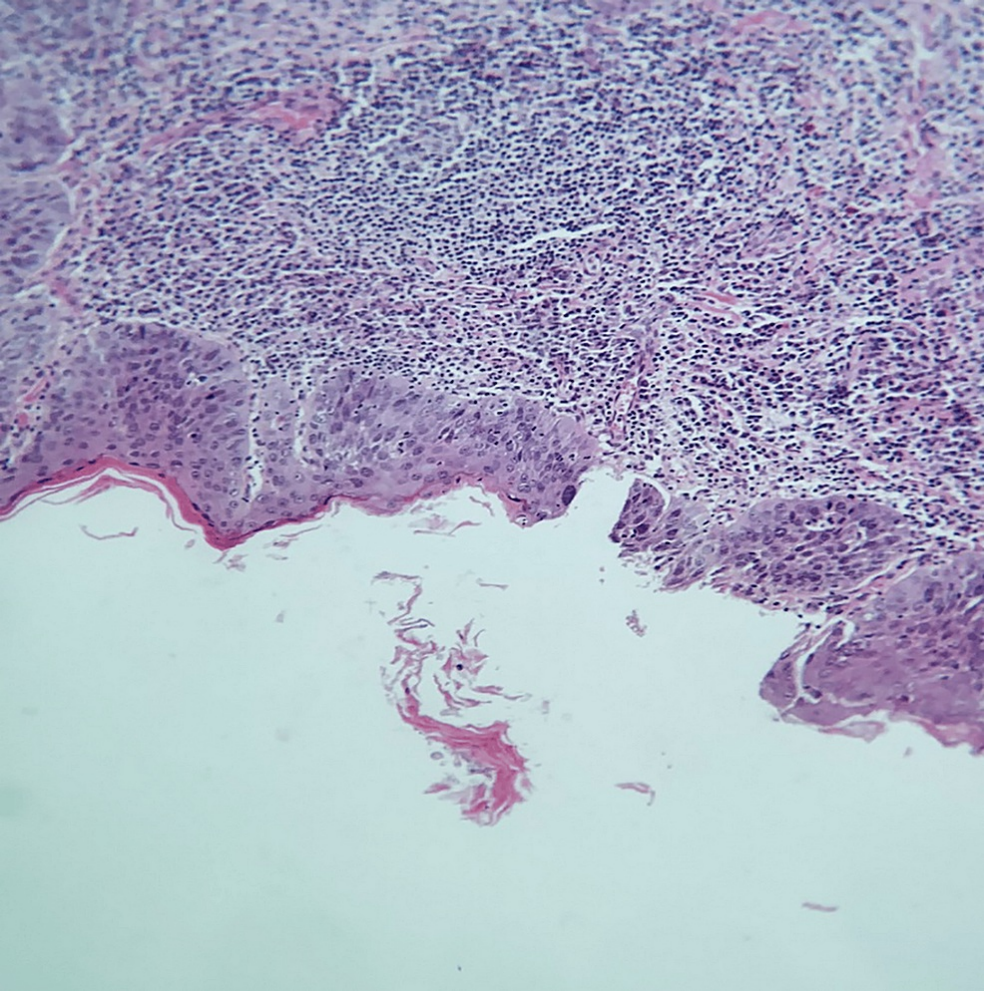
Histopathologic examination revealing a non-specific ulcer.

Cultures of the oral lesions were negative for herpes simplex virus (HSV) type 1 (HSV-1) and HSV type 2 (HSV-2), cytomegalovirus (CMV), and fungus. The rapid plasma regains (RPR) and HIV antibody testing results were non-reactive. The histopathologic examination also confirmed a non-specific ulcer (Figure 3). Disrupted epithelial due to the extensive non-specific inflammatory response was noted. The epithelial layer consists of keratinized stratified squamous epithelium that is uneven and irregular in thickness and disrupted in some areas due to non-specific inflammatory infiltrate constituting mainly lymphocytes. Laboratory investigations returned typical results for liver function, hemoglobin level, platelet count, serum creatinine, serum vitamin B12, and folic acid. However, C-reactive protein (CRP) levels and white blood cell (WBC) count were elevated.

Treatment

**Figure 4:**
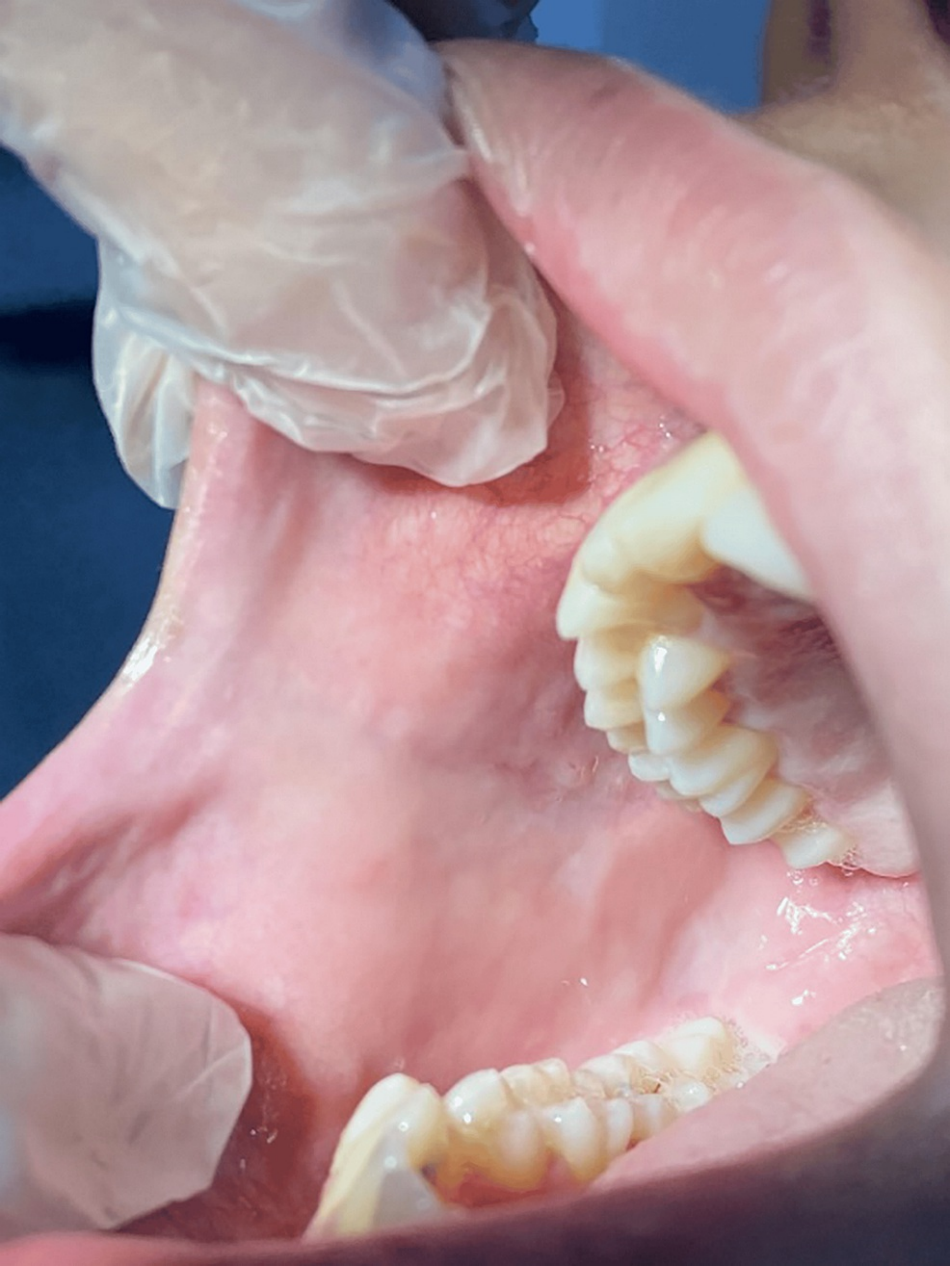
Complete healing of the patient’s ulcers after two weeks with no evidence of recurrences.

After communicating with the patient's physician, the immunosuppressive drug mycophenolate mofetil was stopped due to oral complications. The patient was switched to azathioprine and tacrolimus as a maintenance immunosuppressant regimen. After five days, the patient verbalized a significant reduction in her oral pain, and the size of the oral ulcers decreased considerably, with improved ability to eat and drink. Complete healing of the patient’s ulcers was observed after two weeks. On follow-up of the patient, no recurrences were reported (Figure 4). A signed consent form was obtained from the patient after providing the required information.

## Discussion

Mycophenolate mofetil is an immunosuppressant medicine commonly used as part of an immunosuppressive regimen to avert acute transplant rejection in solid organ or hematopoietic stem cell transplant recipients [9,10]. The mechanism by which mycophenolate mofetil works is blocking purine synthesis by antagonizing the inosine phosphate dehydrogenase enzyme. This consequently leads to a reduction in the proliferation of lymphocytes and thereby accounts for the immunosuppressive property of the medicine [8,11]. Some notable adverse effects of mycophenolate mofetil are nausea, vomiting, diarrhea, dyspepsia, stomach ache, and gastroesophageal reflux [4]. However, oral ulceration secondary to mycophenolate mofetil is rare and, therefore, not well-recognized in healthcare medicine [6-8].

A handful of case reports have been published in the literature regarding the development of oral ulcerations stemming from mycophenolate mofetil among kidney transplant recipients [2,6-8,12-15]. Table 2 summarizes the case report literature on mycophenolate mofetil-induced oral ulcers in kidney transplant recipients. Generally, oral ulcers clinically manifest as painful lesions of diverse sizes and shapes, ranging from shallow bumps to deep ulcers, with surrounding edema and erythema [7]. Presently, a general agreement is that drug-induced oral ulcers are a diagnostic challenge in solid organ transplant recipients; therefore, other causes, such as infectious diseases and cancers, must be omitted [2,6,12,14-15].

**Table 2:** Literature review of mycophenolate mofetil-induced oral ulcers in kidney transplant recipients.

Studies	Age (years)	Gender	Time since kidney transplantation	Areas affected with ulcers	Histopathology features	Immunosuppressive medicines and dosing	Outcome after discontinuation of mycophenolate mofetil
Garrigue et al., 2001 [13]	52	Male	6 months	Mouth and anus	Non-specific inflammatory changes	Cyclosporine = 400 mg/day, prednisolone = 10 mg/day mycophenolate mofetil = 2 g/day	Complete resolution of oral ulcers after 3 weeks of discontinuation of mycophenolate mofetil
Apostolou et al., 2004 [12]	62	Female	3 years	Lingual mucosa and soft palate	Non-specific inflammatory changes	Tacrolimus = 4 mg/day, prednisone = 7.5 mg/day, mycophenolate mofetil = 2 g/day	Complete resolution of oral ulcers after 6 weeks of discontinuation of mycophenolate mofetil
Weng et al.,2011 [15]	23	Male	5 months	Gingiva, oral mucosa, labial mucosa	Biopsy was not performed	Cyclosporine = 500 mg/day, prednisolone = 20 mg/day, mycophenolate mofetil = 2 g/day	Complete resolution of oral ulcers after 6 weeks of discontinuation of mycophenolate mofetil
Philipone et al., 2014 [16]	54	Female	5 months	Upper lip, hard palate, buccal mucosa, alveolar crest	Non-specific inflammatory changes	Tacrolimus = 16 mg/day, mycophenolate mofetil = 2.16 g/day	Complete resolution of oral ulcers after 4 weeks of dose reduction of tacrolimus and mycophenolate mofetil
Salik et al., 2015 [14]	68	Male	8 months	Soft palate	Non-specific inflammatory changes	Tacrolimus = 16 mg/day mycophenolate mofetil = 2 g/day	Complete resolution of oral ulcers after 9 days of discontinuation of mycophenolate mofetil
Plana-Pla et al., 2019 [8]	67	Male	9 months	Tongue, buccal mucosa	Non-specific inflammatory changes	Tacrolimus = 16 mg/day, prednisolone = 5 mg/day, mycophenolate mofetil = 2 g/day	Complete resolution of oral ulcers after 9 days of discontinuation of mycophenolate mofetil
Asare and Gatzke, 2020 [2]	54	Male	7 months	Tongue, esophagus	Non-specific inflammatory changes	Tacrolimus = 8 mg/day, mycophenolate mofetil = 2 g/day	Complete resolution of oral ulcers after 12 days of discontinuation of mycophenolate mofetil
Gaied et al., 2020 [6]	41	Male	5 months	Tongue, buccal mucosa lip	Non-specific inflammatory changes	Tacrolimus = 12 mg/day, prednisone = 10 mg/day, mycophenolate mofetil = 2 g/day	Complete resolution of oral ulcers after 9 days of discontinuation of mycophenolate mofetil
Tenório et al., 2020 [7]	54	Female	11 years	Buccal mucosa, soft palate, tongue, dorsum	Non-specific inflammatory changes	Tacrolimus = 4 mg/day, prednisone = 5 mg/day, mycophenolate mofetil = 1.44 g/day	Complete resolution of oral ulcers within 1 month of dose reduction of tacrolimus and mycophenolate mofetil

It is important to note here that the therapeutic dose of mycophenolate mofetil is up to 3 g/day; however, the research data suggests that the response to this medicine could be individual to kidney transplant recipient based on fluctuation in the dose-response relationship [6,8,12-16]. It is emphasized that the exposure to mycophenolate mofetil must be monitored, and its dosage must be adjusted accordingly for anticipated clinical outcomes [17]. In our patient, the dose was above the limit, i.e., 2 g every 12 h per oral, which could have led to inflammation of the oral mucosa and, thereby, mycophenolate mofetil-induced oral ulcer.

For the current case, we ruled out the possibility of infection as a cause of oral ulcers because cultures of the oral lesions were negative for HSV-1 and HSV-2, CMV, and fungus, and RPR and HIV antibody testing results were non-reactive. Immunosuppression substantially increases the vulnerability of infections among kidney transplant recipients and can cause primary infection or reemergence of previous dormant pathogens. Generally, infections caused by HSV-1, HSV-2, and CMV in immunocompromised patients are disseminated and often complicated [18-19]. Furthermore, oral ulcers triggered by infectious etiology, especially originating from viruses like CMV, have diverse clinical manifestations, such as small eruptions, edema, erythema, pain, and deep ulcers. In such scenarios, IgM anti-CMV IgM antibodies are positive and IgG antibody levels are raised [7].

Histopathological analysis assists in identifying the origin of oral ulcers. In the present case, histopathologic examination confirmed a non-specific ulcer for viral causes like CMV, chronic inflammatory exudate inflamed endothelial cells, and numerous nuclear and cytoplasmic inclusion bodies (often referred to as owl’s eyes) are regularly witnessed [20]. This was not the case in the present patient, prompting us to disregard the likelihood of viral origin.

Scientific literature suggests two conceivable clinical measures when the diagnosis of mycophenolate mofetil-induced oral ulcers is made. First, to switch from mycophenolate mofetil to other medicine with no or lesser known toxicity related to oral ulcers [6,8,12-15]. Enteric-coated mycophenolate sodium has been reported as a possible replacement for mycophenolate mofetil [17]. The second way is either withdrawal or reducing the dose of mycophenolate mofetil. This decision solely depends on the patient’s clinical situation, allograft rejection history, and nephrology consultants’ expertise [6-8,12-16].

The scientific literature confirms that mycophenolate mofetil and tacrolimus, in combination, are superior in reducing acute rejection and mortality among kidney transplant recipients in contrast to other immunosuppressive combinations [21]. On the other hand, previous research reports have shown the concomitant use of mycophenolate mofetil and tacrolimus to induce oral ulcers [12,14-16]. However, in the present case, the patient had no history of kidney allograft rejection, and mycophenolate mofetil was the most likely cause of oral ulcer; therefore, we decided to switch to mycophenolate mofetil. This resulted in complete resolution immediately after discontinuation of mycophenolate mofetil. There is a risk of developing complications in patients undertaking both tacrolimus and mycophenolate mofetil (MMF). However, there is no clear understanding of how immunosuppressive agent combination induces the risk of developing oral ulcers.

While the exact mechanisms by which mycophenolate mofetil induces oral ulcerations in kidney transplant recipients are yet to be unearthed, two possible assumptions have been suggested. First, the etiology of oral ulcers could lie in opportunistic infections caused by bacteria, viruses, and fungi due to extreme immunosuppression of the patient [22]. However, this could be the less likely cause as evidence reports otherwise. Besides reducing immunosuppression, introducing antiviral medicines should resolve oral ulcers if the virus is the most likely pathologic [15]. In fact, efforts were made to treat mycophenolate mofetil-induced oral ulcers with anti-viral medicines; however, to no avail [12-13]. The second assumption is related to the anti-proliferative and cytotoxic properties of mycophenolic acid, which is the active compound of mycophenolate mofetil, on the oral mucosa [13,23]. At the very least, this hypothesis stays true as mycophenolate mofetil-induced oral ulcers have been shown to disappear after the withdrawal of mycophenolate mofetil completely. Our finding also agrees with earlier reports [2,6,8,12-15].

All the case reports regarding mycophenolate mofetil-induced oral ulcers in kidney transplant recipients have reported the mycophenolate mofetil dose of approximately 1-2 g/day. It was further noted that the oral ulcers resolved as soon as the mycophenolate mofetil was discontinued [2,6,8,12-15], or, in a few cases, when the dose was reduced [7,16]. Complete resolution of the oral ulcers was witnessed within six weeks at the maximum [12,15]. Notwithstanding, mycophenolate mofetil remains the rarest of the etiologies of oral ulcers. Nonetheless, it is a sine qua non to exclude all potential causes like bacteria, viruses, and fungi infections. Moreover, a histopathologic examination must be conducted to eliminate other illnesses.

## Conclusions

In conclusion, oral ulcerations in a kidney transplant recipient can occur from various etiologies. Therefore, diagnosing mycophenolate mofetil-induced oral ulcerations requires excluding other potential differential diagnoses like infectious diseases instigated by CMV, HIV, HSV, and some cancers. In the present case, the oral ulcers resolved immediately after discontinuing the mycophenolate mofetil. Mycophenolate mofetil rarely induces oral ulcerations in kidney transplant recipients; however, physicians should be aware of it. Withdrawal of mycophenolate mofetil or switching to other medicine must be considered when a patient with a kidney transplant history complains of oral ulcers and a negative workup for other etiologies. At the same time, it is essential to continuously monitor the patient after medicine switching so that a balance can be attained between immunosuppression to circumvent allograft rejection and secondary adverse effects. Further studies (i.e., clinical trials) are recommended to confirm these results.
